# A Study of Adrenal Incidentaloma-Related Hormonal Assays After First Integration of the Diagnosis Within Primary Healthcare

**DOI:** 10.3390/diseases13060169

**Published:** 2025-05-26

**Authors:** Oana-Claudia Sima, Mihai Costachescu, Ana Valea, Mihaela Stanciu, Ioana Codruta Lebada, Tiberiu Vasile Ioan Nistor, Mihai-Lucian Ciobica, Claudiu Nistor, Mara Carsote

**Affiliations:** 1 PhD Doctoral School of “Carol Davila” University of Medicine and Pharmacy, 010825 Bucharest, Romania; oana-claudia.sima@drd.umfcd.ro; 2 Department of Radiology and Medical Imaging, Fundeni Clinical Institute, 022328 Bucharest, Romania; mihai.costachescu@drd.umfcd.ro; 3 Department of Endocrinology, “Iuliu Hatieganu” University of Medicine and Pharmacy, 400012 Cluj-Napoca, Romania; 4 Department of Endocrinology, County Emergency Clinical Hospital, 400347 Cluj-Napoca, Romania; 5 Department of Endocrinology, “Lucian Blaga” University of Sibiu, 550024 Sibiu, Romania; codruta.lebada@ulbsibiu.ro; 6 Department of Endocrinology, Clinical County Emergency Hospital, 550245 Sibiu, Romania; 7 Medical Biochemistry Discipline, “Iuliu Hatieganu” University of Medicine and Pharmacy, 400347 Cluj-Napoca, Romania; tiberiu.nistor@umfcluj.ro; 8 Department of Internal Medicine and Gastroenterology, “Carol Davila” University of Medicine and Pharmacy, 020021 Bucharest, Romania; lucian.ciobica@umfcd.ro; 9 Department 4—Cardio-Thoracic Pathology, Thoracic Surgery II Discipline, “Carol Davila” University of Medicine and Pharmacy, 0505474 Bucharest, Romania; claudiu.nistor@umfcd.ro; 10 Department of Endocrinology, “Carol Davila” University of Medicine and Pharmacy, 020021 Bucharest, Romania; carsote_m@hotmail.com

**Keywords:** primary health, adrenal, tumor, imaging, hormone, endocrine, assay, computed tomography, ACTH, incidentaloma

## Abstract

Background: Adrenal incidentalomas are detected in various medical and surgical healthcare departments, including primary healthcare. One up to three out of ten individuals confirmed with nonfunctioning adrenal incidentalomas (NFAs) actually present a mild autonomous cortisol secretion (MACS), which is distinct from Cushing’s syndrome. Objective: We aimed to assess the cortisol secretion in newly detected adrenal incidentalomas in patients who were referred by their primary healthcare physician upon accidental detection of an adrenal tumor at abdominal computed tomography (CT) scan that was performed for unrelated (non-endocrine) purposes. Methods: This retrospective study included adults diagnosed with an adrenal incidentaloma via CT during the previous 3 months. Inclusion criteria: age ≥ 40 years (y). A triple stratification of exclusion criteria involved: (1) Clinical aspects and medical records such as active malignancies or malignancies under surveillance protocols, subjects under exogenous glucocorticoid exposure (current or during the previous year), or suggestive endocrine phenotypes for any hormonal ailment; (2) Radiological appearance of suspected/confirmed (primary or secondary) adrenal malignancy, adrenal cysts, or myelolipomas; (3) Endocrine assays consistent with active endocrine tumors. Protocol of assessment included baseline ACTH, morning plasma cortisol (C-B), cortisol at 6 p.m. (C-6 pm), and after 1 mg dexamethasone suppression testing (C-1 mg-DST), 24-h urinary free cortisol (UFC), and a second opinion for all CT scans. MACS were defined based on C-1 mg-DST ≥ 1.8 and <5 µg/dL (non-MACS: C-1 mg-DST < 1.8 µg/dL). Results: The cohort (N = 60, 78.33% female; 60.72 ± 10.62 y) associated high blood pressure (HBP) in 66.67%, respectively, type 2 diabetes (T2D) in 28.37% of the patients. Females were statistically significantly older than males (62.40 ± 10.47 vs. 54.62 ± 9.11 y, *p* = 0.018), while subjects with unilateral vs. bilateral tumors (affecting 26.67% of the individuals) and those with MACS-positive vs. MACS-negative profile had a similar age. Body mass index (BMI) was similar between patients with unilateral vs. bilateral incidentalomas, regardless of MACS. Patients were divided into five age groups (decades); most of them were found between 60 and 69 years (40%). Left-gland involvement was found in 43.33% of all cases. The mean largest tumor diameter was 26.08 ± 8.78 mm. The highest rate of bilateral tumors was 46.67% in the 50–59 y decade. The rate of unilateral/bilateral and tumor diameters was similar in females vs. males. The MACS-positive rate was similar in females vs. males (23.40% vs. 23.08%). A statistically significant negative correlation (N = 60) was found between BMI and C-B (r = −0.193, *p* = 0.03) and BMI and UFC (r = −0.185, *p* = 0.038), and a positive correlation was found between C-B and C-6 pm (r = 0.32, *p* < 0.001), C-B and UFC (r = 0.226, *p* = 0.011), and C-6 pm and C-1 mg-DST (r = 0.229, *p* = 0.010), and the largest tumor diameter and C-1 mg-DST (r = 0.241, *p* = 0.007). Conclusions: Adrenal incidentalomas belong to a complex scenario of detection in the modern medical era, requiring a multidisciplinary collaboration since the patients might be initially detected in different departments (as seen in the current study) and then referred to primary healthcare for further decision. In these consecutive patients, we found a higher female prevalence, a MACS rate of 23.33%, regardless of uni/bilateral involvement or gender distribution, and a relatively high rate (than expected from general data) of bilateral involvement of 26.67%. The MACS-positive profile adds to the disease burden and might require additional assessments during follow-up and a protocol of surveillance, including a tailored decision of tumor removal. The identification of an adrenal incidentaloma at CT and its hormonal characterization needs to be integrated into the panel of various chronic disorders of one patient. The collaboration between endocrinologists and primary healthcare physicians might improve the overall long-term outcomes.

## 1. Introduction

Accidentally detected adrenal masses may be found at thoracic, abdominal, or pelvic imaging assessment (e.g., computed tomography (CT) scans), and an increasing rate of their diagnosis has been found in the modern medical era [1,2,3]. Moreover, there is an age-related incidence, as well, with most of the patients being adults over 40 years [4,5,6]. Incidentally diagnosed adrenal tumors (adrenal incidentalomas) may be found in apparently healthy individuals or in those who display unrelated clinical elements such as kidney stones, back pain, etc., or who underwent an imaging procedure due to recent trauma or followed a checkup protocol for a non-adrenal malignancy [7,8,9].

Under these heterogeneous circumstances, adrenal incidentalomas are actually detected in various medical and surgical healthcare departments, including primary healthcare [10,11,12]. The next step after the imaging confirmation of such a tumor is to refer the patient to an endocrine unit and to assess the hormonal panel [13,14,15].

Generally, after the preliminary radiological evaluation, most patients do not present tumors with suspected features for a malignancy (such as a large size (e.g., a diameter over 4 cm), irregular shape, local invasion, spreading, etc.) since adrenal malignancy represents an exceptional finding [16,17,18]. Furthermore, endocrine evaluation is mandatory, and one tumor might be functioning or nonfunctioning. From an epidemiologic perspective, most of them are hormonally inactive, namely nonfunctioning adrenal adenomas (NFAs) [19,20,21]. However, recent data showed that one up to three out of ten individuals confirmed with NFAs actually present a mild autonomous cortisol secretion (MACS) which is distinct from Cushing’s syndrome [22,23,24]. Following the NFA ± MACS confirmation, the patients will be conservatively managed, and long-term surveillance is required. Only a selected subgroup of patients becomes surgery candidates at the same point across the lifespan, but this is not the standard care in cases of NFAs [25,26,27].

### Objective

We aimed to assess the ACTH-cortisol secretion in newly detected adrenal incidentalomas in patients who were referred by their primary healthcare physician upon accidental detection of the adrenal tumor at an abdominal CT scan that was performed for unrelated (non-endocrine) purposes.

## 2. Patients and Methods

Study design: this was a retrospective, cross-sectional, observational, bi-centric, real-life, one-year study (between January 2023 and December 2023).

Study population: consecutive adult cases who had a primary detection of an adrenal tumor amid a CT scan (that was performed for non-endocrine purposes) and were confirmed with an adrenal incidentaloma (NFA ± MACS). The patients were referred for an endocrine checkup by their primary healthcare physician.

Inclusion criteria: age of 40 years or older; unilateral or bilateral adrenal tumors that were diagnosed for the first time at CT scan during the prior 3 months; lack of clinical features/phenotype that might be suggestive of an endocrine disease, including an adrenal disorder.

Exclusion criteria involved a triple stratification as follows:

1. clinical aspects and medical records (active malignancies or malignancies under surveillance protocols; subjects under exogenous glucocorticoids exposure (current or during the previous year); suggestive endocrine phenotype for any hormonal ailment)
2. radiological appearance (subjects with distinct imaging features at CT scan such as suspected/confirmed primary or secondary adrenal malignancy, adrenal cysts, or myelolipomas)
3. endocrine assays consistent with a diagnosis of pheochromocytoma, Conn’s syndrome, or Cushing’s syndrome

Study protocol: the patients were initially detected with adrenal incidentalomas after performing a CT scan for non-endocrine aspects such as trauma, thoracic–abdominal CT for a previous pulmonary infection, back pain, or kidney stones. Upon detection of an adrenal incidentaloma in different non-endocrine departments, the primary healthcare physician referred them to an endocrine unit where they had a 48-h hospitalization (inpatients) to assess the adrenal hormonal profile. Of note, the CT scan was performed at a maximum of 3 months before the endocrine assessment.

Data collection included the age, the medical history, e.g., prevalent high blood pressure and type 2 diabetes mellitus, as well as the calculation of body mass index (BMI) in kg/sqm.

CT scans were re-analyzed (second opinion) by a trained radiologist (M.K.) and confirmed unilateral/bilateral adrenal incidentalomas and the size in terms of the largest diameter of each tumor (mm) that has been used in the final analysis, and excluded the imaging features that were mentioned at exclusion criteria. Notably, if an adrenal malignancy (e.g., metastases) could not be clearly ruled out, the patient was excluded from the study.

Endocrine investigations in the study included baseline plasma ACTH (adrenocorticotropic hormone), baseline morning plasma (fasting) cortisol (C-B), plasma cortisol at 6 p.m. (C-6 pm), 24-h urinary free cortisol (UFC), and second-day plasma cortisol after the administration of 1 mg dexamethasone at 11 p.m. according to the suppression test (C-1 mg-DST). The MACS-positive category was defined based on C-1 mg-DST equal or above 1.8 µg/dL, respectively, below 5 µg/dL (non-MACS included C-1 mg-DST < 1.8 µg/dL). For exclusion criteria, patients underwent plasma metanephrine/normetanephrine assays and aldosterone/renin ratio, and only those patients with normal results were included. Also, individuals with C-1 mg-DST value of ≥ 5 µg/dL were excluded, regardless of whether the clinical phenotype was highly suggestive of Cushing’s syndrome.

**Figure 1:**
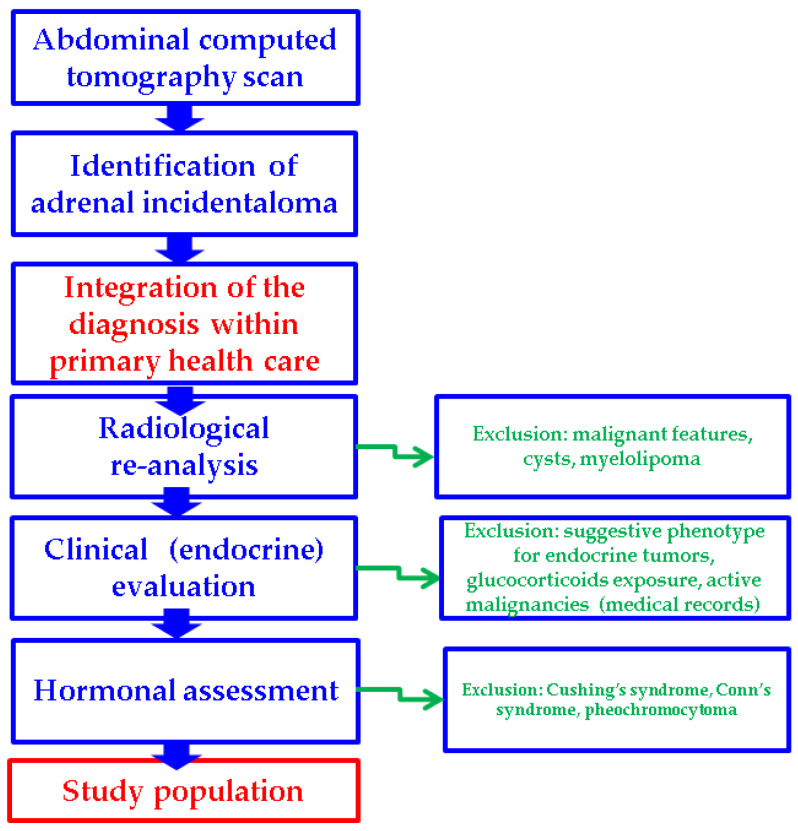
Study protocol according to our methods.

Additionally, four types of subgroup analyses were performed: patients with unilateral versus (vs.) bilateral adrenal incidentalomas, the tumor features in female vs. male populations, and subjects with MACS-positive vs. MACS-negative incidentalomas, as well as an age-decade analysis (Figure 1).

Statistical analysis: The distribution patterns of continuous variables were examined both visually and through the Kolmogorov–Smirnov normality test to determine whether they followed a Gaussian distribution. For descriptive analysis, normally distributed variables were expressed as mean ± standard deviation (SD), while non-normally distributed variables were summarized using quartiles (first quartile/Q1, median/Q2, and third quartile/Q3). Either the chi-squared test or Fisher’s exact test was applied, depending on data characteristics, in order to investigate the relationship between categorical variables. Comparisons between two independent groups were performed using the Student’s *t*-test for variables with a normal distribution, whereas the Mann–Whitney U-test was employed for non-normally distributed data. For comparisons involving multiple groups, the Kruskal–Wallis test was applied for non-parametric data. The association between numerical variables was quantified using Kendall’s Tau correlation coefficient. A *p*-value below 0.05 was considered indicative of statistical significance. The statistical evaluation of the data was carried out using Excel 16.95 (Microsoft, Redmond, WA, USA) and SPSS 29.0.2.0 (SPSS, Inc., Chicago, IL, USA).

Ethical aspects: The included patients signed the informed consent during hospitalization according to each hospital protocol. Retrospective data collection was approved by the local Ethics Committees of the hospitals.

## 3. Results

### 3.1. Baseline Clinical and Hormonal Features

A total of 60 patients with adrenal incidentalomas were included (78.33% were female; the age at diagnosis was 60.72 ± 10.62 years). A prevalent diagnosis of hypertension was found in 66.67% of them, and 28.37% were confirmed with type 2 diabetes (Table 1).

**Figure 2:**
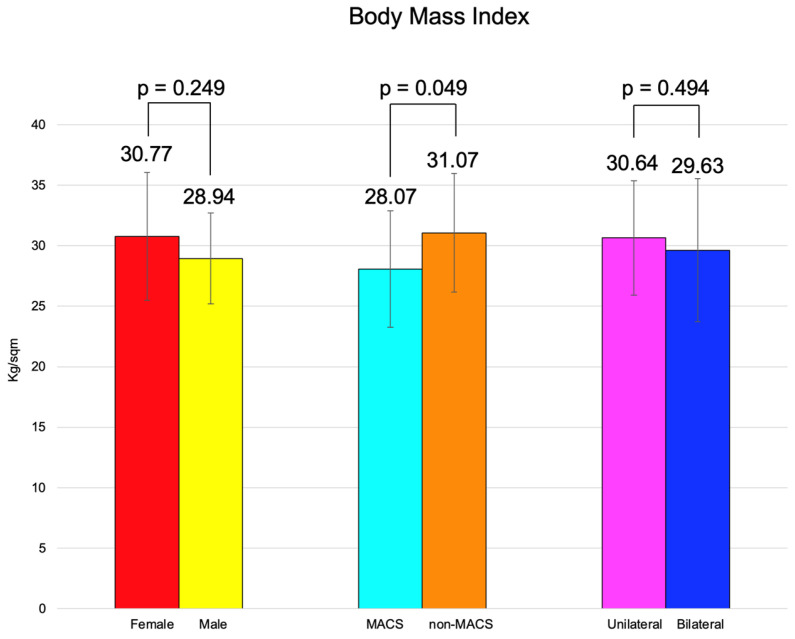
Clustered bar charts showing mean age in the studied subgroups (with 95% confidence interval).

BMI was similar between females and males, between the patients with MACS vs. non-MACS, and the subjects with unilateral vs. bilateral tumors (Figure 2).

Bilateral incidentalomas were found in 26.67% of the individuals. A total of 30.00% of the patients had a tumor on the right adrenal gland and 43.33% of them on the left adrenal gland, with an average largest tumor diameter of 26.08 ± 8.78 mm (Table 2).

**Figure 3:**
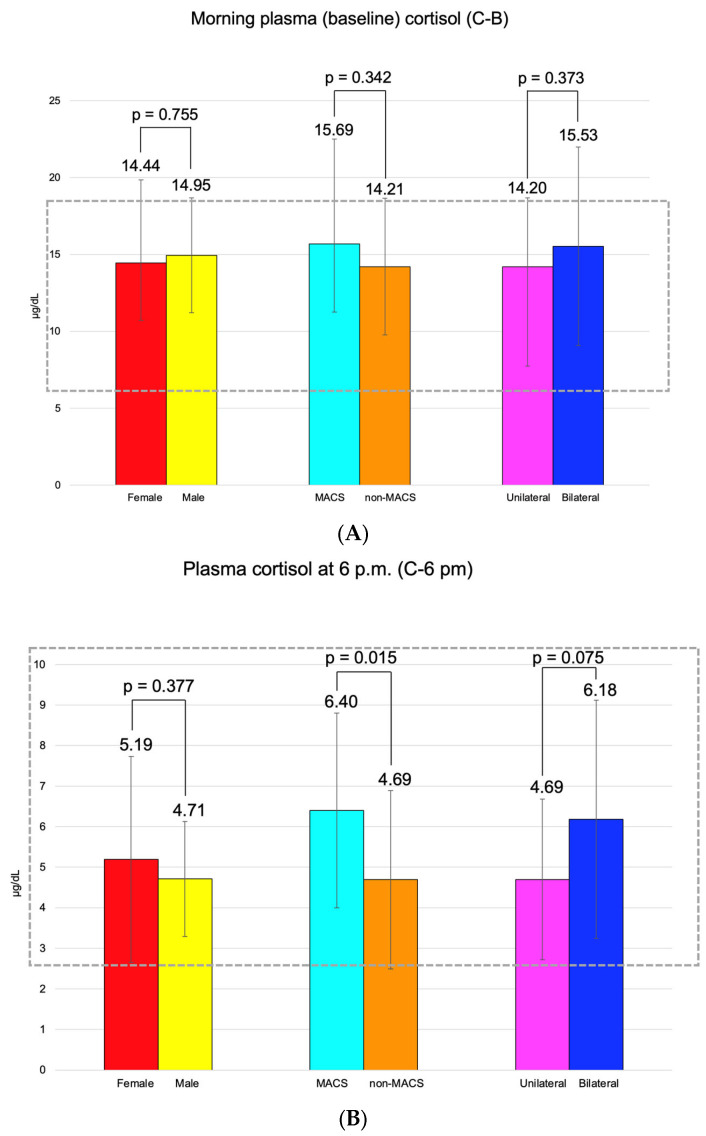

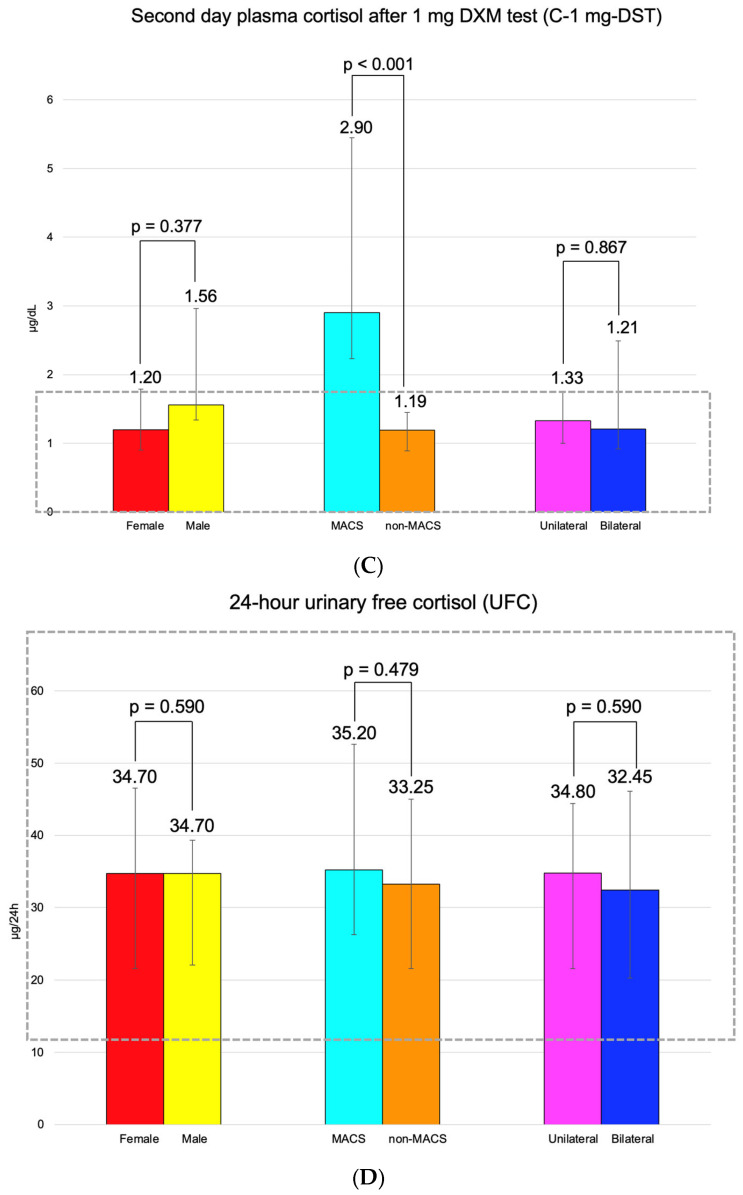
(**A**). Clustered bar charts showing mean morning plasma (baseline) cortisol levels in the studied subgroups (with a 95% confidence interval). (**B**). Clustered bar charts showing mean plasma cortisol at 6 p.m. levels in the studied subgroups (with a 95% confidence interval). (**C**). Clustered bar charts showing median second-day plasma cortisol after 1 mg dexamethasone suppression test levels in the studied subgroups (with 95% confidence interval). (**D**). Clustered bar charts showing median 24-h urinary free cortisol levels in the studied subgroups (with 95% confidence interval).

C-B had an average of 14.55 ± 5.07 μg/dL (and it was found to be similar between the three pairs of mentioned subgroups). C-6 pm was of 5.09 ± 2.34 μg/dL; median C-1 mg-DST was 1.32 (0.10, 1.79) μg/dL, and UFC was 34.70 (21.60, 45.85) μg/24 h (Figure 3).

### 3.2. Age-Decade Analysis

The patients were divided into five age groups. Most of them were found in the 60–69 years subgroup (40%) (Table 3).

**Figure 4:**
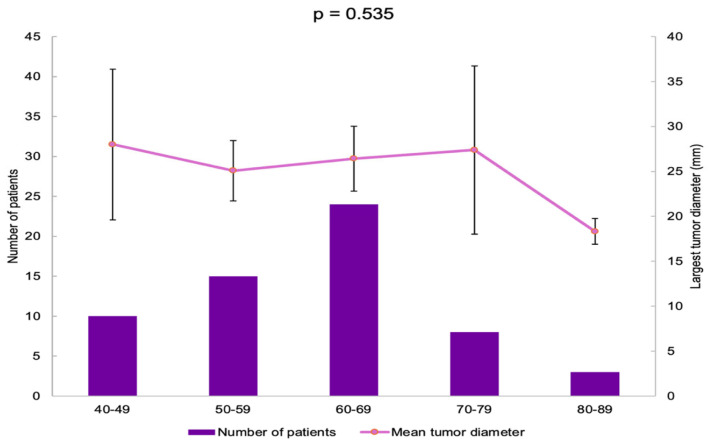
Bar chart showing the number of patients in each age group and line chart showing mean largest tumor diameter in each age group (error bars showing 95% confidence interval).

The largest tumor diameter was found to be 28.00 ± 11.72 mm in the 40–49 years group, and it was similar among all the age groups (*p* = 0.535) (Figure 4).

**Figure 5:**
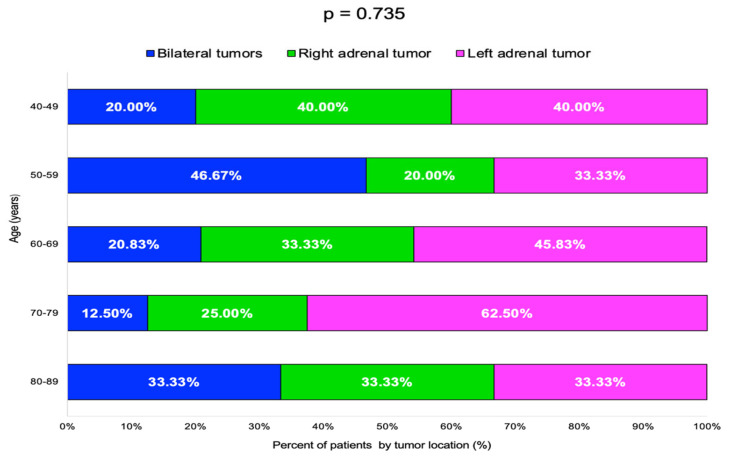
Bar chart showing percentage of patients by adrenal incidentalomas location and age groups (N = 60).

The highest rate of bilateral tumors was 46.67% in the 50–59 years decade, while for single incidentalomas, the rate of right/left-gland involvement varied without statistical significance (Table 4, Figure 5).

**Figure 6:**
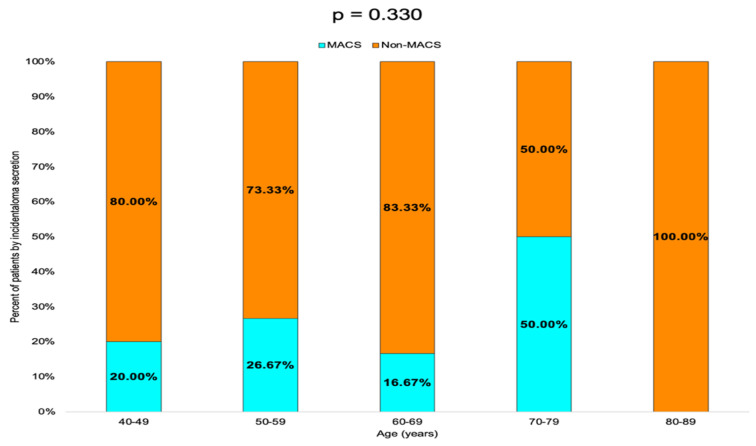
Bar chart showing the percentage of patients with MACS-positive vs. MACS-negative tumors by age group.

Non-MACS profile was found in 80.00% of the patients in the 40–49 years age group, 73.33% in the 50–59 years group and 83.33% in the 60–69 years group. In the 70–79 years group, the rate of MACS-positive and non-MACS tumors was equal (50.00%), while in the 80–89 years group, 100.00% of the tumors were non-MACS (Table 5, Figure 6).

### 3.3. Correlations Between the Adrenal Incidentaloma Features

**Figure A1:**
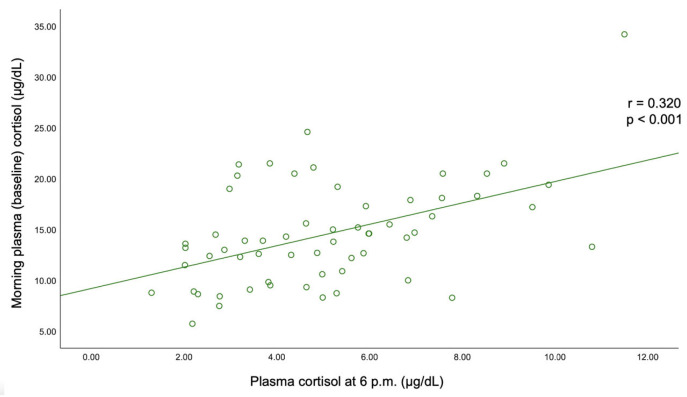
Correlation between the morning plasma (baseline) cortisol and plasma cortisol at 6 p.m.

**Figure A2:**
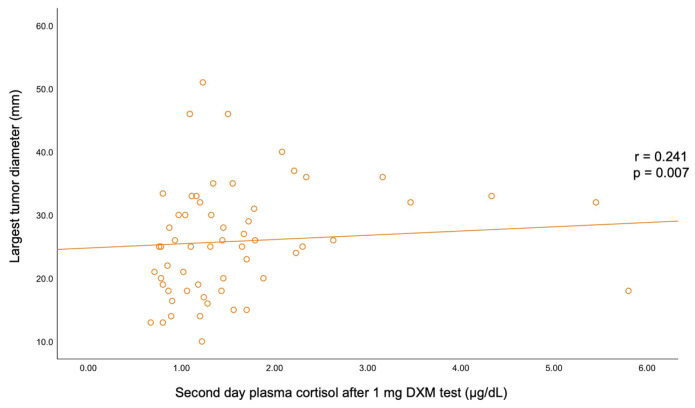
Correlation between the largest tumor diameter and second-day plasma cortisol after 1 mg dexamethasone testing.

A statistically significant negative correlation was found between BMI and C-B (r = −0.193, *p* = 0.03) and between BMI and UFC (r = −0.185, *p* = 0.038). C-B positively correlated with C-6 pm (r = 0.32, *p* < 0.001) and with UFC (r = 0.226, *p* = 0.011). A positive correlation was also found between C-6 pm and C-1 mg-DST (r = 0.229, *p* = 0.01). The largest tumor diameter statistically significantly correlated with C-1 mg-DST (r = +0.241, *p* = 0.007) (Table 6, Figure A1 and Figure A2).

### 3.4. Gender Analysis

The age of adrenal incidentaloma diagnosis was statistically significantly higher in females compared to the male population (62.40 ± 10.47 vs. 54.62 ± 9.11 years, *p* = 0.018) (Table 7).

Unilateral/bilateral tumor rate were similar between females and males (*p* = 0.741), as well as the incidentaloma size, as reflected by the largest tumor diameter (*p* = 0.357), and the hormonal panel (C-B, C-6 pm, C-1 mg-DST, and UFC). The rate of MACS was similar between females (23.40%) and males (23.08%) (*p* = 0.980) (Table 8).

### 3.5. Unilateral Versus Bilateral Incidentalomas

Age and BMI were similar among the patients with unilateral vs. bilateral adrenal incidentalomas (*p* = 0.407, respectively, *p* = 0.494) (Table 9).

The largest tumor diameter was not statistically significantly different between patients with unilateral vs. bilateral tumors (*p* = 0.408). C-B and C-1 mg-DST were similar in subjects with unilateral vs. bilateral tumors. The rate of MACS was 20.45% and 31.25% in unilateral vs. bilateral tumors, respectively (Table 10).

### 3.6. MACS-Positive Versus MACS-Negative Profile in Adrenal Tumors

BMI of the subjects with MACS was statistically significantly lower vs. subjects without MACS (28.07 ± 4.83 vs. 31.07 ± 4.92 kg/sqm, *p* = 0.049) (Table 11).

Adrenal incidentalomas with a MACS-positive profile had an increased largest diameter of 30.14 ± 6.81 mm vs. MACS-negative of 24.84 ± 9.00 mm (*p* = 0.047). C-6 pm and C-1 mg-DST were statistically significantly elevated in subjects with MACS vs. non-MACS subgroups (*p* = 0.015 and *p* < 0.001, respectively), while C-B had similar values (*p* = 0.342) (Table 12).

## 4. Discussion

### 4.1. Demographic Features and Clinical Picture in Subjects with Adrenal Incidentalomas

**Figure 7:**
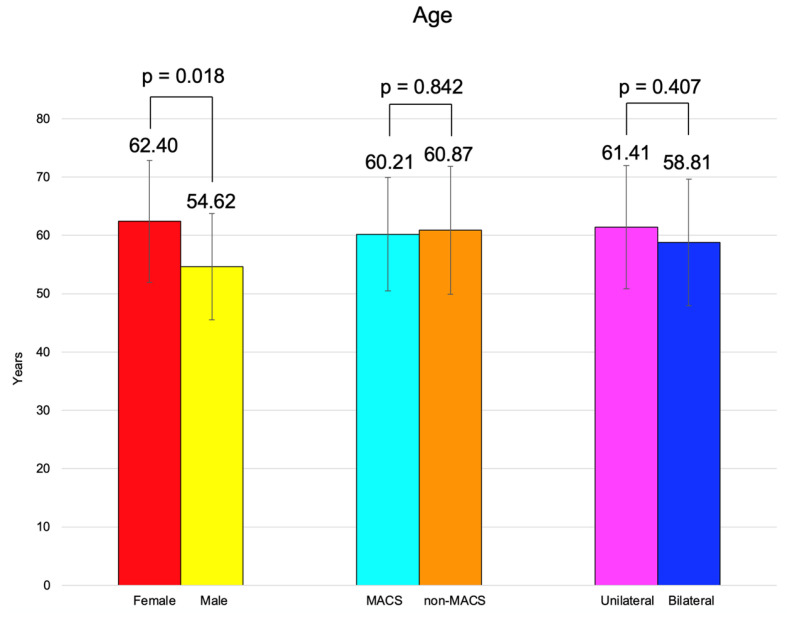
Overview of the age analysis: clustered bar charts showing mean age in the studied subgroups (with 95% confidence interval).

We analyzed 60 adult cases (mean age of 60.72 years) with female predominance (78.33%). These patients were referred from by primary healthcare physicians after they had had an abdominal CT scan for non-endocrine purposes and were identified with at least one adrenal incidentaloma. Generally, this scenario requires a multidisciplinary team collaboration since the patients might come from different departments (as seen in the current study) [28,29,30]. These consecutive patients, according to the inclusion/exclusion criteria, provided a confirmation of previous data with respect to the peak age incidence in adults a female-to-male ratio of 3.6 [31]. The age of incidentaloma diagnosis was statistically significantly higher in females compared to males (62.40 ± 10.47 vs. 54.62 ± 9.11 years, *p* = 0.018). It was similar between subjects with unilateral vs. bilateral tumors and with MACS-positive vs. MACS-negative profiles. The age-group (decades) analysis showed that 40% of them were found to be between 60 and 69 years (Figure 7).

Cortisol excess, even mild and not causing the traditional picture of Cushing’s syndrome, might be connected to the development of different ailments such as hypertension (which was found to be prevalent in 66.67% of the patients in this cohort), type 2 diabetes (with a prevalence of 28.37% in our study), hyperlipidemia, or osteoporosis [32,33,34]. With respect to these complications, we did not have enough data to introduce them in the final results. Also, BMI was similar between the patients with unilateral vs. bilateral adrenal incidentalomas, regardless of MACS. Furthermore, a statistically significant negative correlation was found between BMI and C-B (r = −0.193, *p* = 0.030) and between BMI and UFC (r = −0.185, *p* = 0.038). Of note, hypertension and type 2 diabetes were similar according to gender analysis. In this specific matter, we mention a retrospective study from 2024 in (non-MACS) NFA (N = 99 subjects, 62.6% were females) and MACS (N = 89 individuals, 64% were females) that showed a cross-sectional (baseline) report with a similar rate for the clinical features and complications between females and males in each tumor category [4].

### 4.2. Adrenal Tumor Imaging Assessment Amid CT Scan

Globally, 90% of the accidentally detected adrenal tumors are considered benign (adenomas), and one third of them might show active endocrine secretion. Modern radiomics approaches include prediction models based on multi-phase enhanced CT images in addition to the clinical characteristics that might help the distinction between different types of adrenal tumors such as aldosteronoma, NFAs, cortisol-producing adenomas, etc. [1]. Some authors have even suggested that CT radiomic analysis in unenhanced CT might serve as a screening tool for further referring patients for an endocrine evaluation regarding their MACS profile [18].

However, the practical implementation of such estimation algorithms largely varies with the medical center, and there are still areas of uncertainty. In this study, bilateral incidentalomas were found in 26.67% of the individuals; the highest rate of bilateral tumors was 46.67% in the group aged between 50 and 59 years. Unilateral analysis showed a left side involvement in 43.33% of all cases; the rate of left/right adrenal lesions varied within age decade without statistical significance. The mean largest tumor diameter was 26.08 ± 8.78 (median (IQR) of 25.50 (19.00, 32.00) mm) and was statistically significantly positively correlated with C-1 mg-DST (r = 0.241, *p* = 0.007). The rate of unilateral/bilateral tumors and the tumor diameters were similar in women vs. men. We mention a retrospective study over a decade in adrenal incidentalomas (N = 384 patients, 64% were females, and 90.6% of the tumors showed benign features) that found a mean tumor diameter of 20 mm [31].

### 4.3. Hormonal Profile in Adrenal Incidentalomas: Focus on MACS

Unrecognized endogenous hypercortisolemia, despite not being clear Cushing’s syndrome, may involve negative cardio-metabolic and osseous effects in the long term [13,35,36]. Overall, 23.33% of the tumors were MACS-positive according to the specific endocrine evaluation (cut-offs based on C-1 mg-DST), and the prevalence of a MACS-free profile was up to 80–between 80 and 100% in some age decades. As with our cohort, one study (published in 2025) of 31 patients diagnosed with adrenal incidentalomas (68% were female, mean age of 55 ± 16.2 years) found that 17% of the tumors showed a MACS-positive profile [2].

Moreover, the hormonal panel (ACTH, C-B, C-6 pm, C-1 mg-DST, and UFC), as well as the rate of MACS positivity (23.40% vs. 23.08%; *p* = 0.980) was similar between females and males. Among the patients with a MACS-positive profile, 64.29% had HBP (vs. 67.4% in non-MACS), but this difference was not statistically significant. In contrast to the expected results, the BMI and type 2 diabetes rates showed borderline significance for being lower in MACS. This might be explained by the fact that obesity and glucose profile anomalies represent a multifactorial issue underlying genetic, epigenetic, and environmental contributors and not a simple consequence of a mild cortisol anomaly [37,38].

Moreover, screening with an oral glucose tolerance test was not systematically performed in non-diabetic subjects, and this may bring a bias of underdiagnosed abnormal glucose status. For instance, one recent study published in 2025 found a higher incidence of diabetes in MACS vs. NFAs (35% vs. 20%), as well as hypertension (60% vs. 45%) [22]. Another study conducted by Han et al. [15] in 64 patients with autonomous cortisol secretion, including 11 of them with MACS and 34 individuals with NFAs, showed a correlation between glucose variability and C-1 mg-DST in each subgroup, which proved that metabolic complications should be taken into consideration, including MACS-negative subjects, despite the fact that a lower rate of glycemic anomalies is expected in NFAs/MACS than that found in clinically manifested Cushing’s syndrome [15].

**Figure 8:**
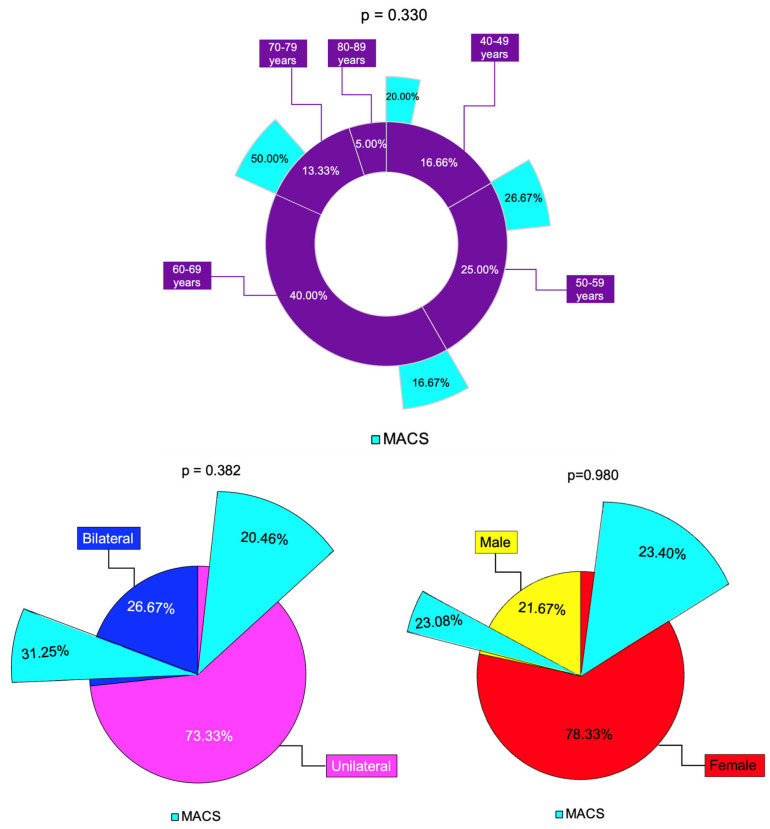
Synopsis of MACS profile among analyzed subgroups: multi-level doughnut chart showing the percentage of patients by age group (magenta) and percentage of patients with MACS-positive profile (turquoise) in each age group; the percentage of unilateral (pink) and bilateral (blue) adrenal incidentalomas, and percentage of patients with MACS-positive tumors (turquoise) in each group; the percentage of females (red) and males (yellows) and percentage of patients with MACS-positive incidentalomas (turquoise) in each group (N = 60).

Some authors have suggested that bilateral adrenal involvement represents an independent predictor of MACS [22]. In this study, bilateral tumors were found in 35.71% of MACS-positive vs. 23.91% in the non-MACS subgroup (*p* = 0.382). The largest diameter was higher in MACS-positive vs. MACS-negative (median (Q1, Q3) of 32.00 (24.75, 36.00) vs. 25.00 (18.00, 30.00), *p* = 0.047) and the value of C-6 pm was statistically significantly higher (6.40 ± 2.40 vs. 4.69 ± 2.20 µg/dL, *p* = 0.015), while the other hormonal assays (except, as expected, for dexamethasone testing results) were similar (Figure 8).

Hormonal parameters might reflect not only the endocrine insights but also other potential risks of cardio-metabolic impairment. For instance, in a recent study in 80 patients with NFAs and 80 (non-NFSAs) controls, an increased Framingham Risk Score was associated with C-1 mg-DST levels [19]. According to our results, a statistically significant positive correlation was also found between C-6 pm and C-1 mg-DST (r = 0.229, *p* = 0.01), which might add to the cortisol excess evaluation, despite not being a mandatory assay according to the latest guidelines for MACS identification [5,6].

### 4.4. From Disease Burden to Healthcare System Integration of Patients with Adrenal Incidentalomas

The impact of even mild cortisol excess is reflected by a complex panel of comorbidities, especially in a long-term outcome and a worsened quality of life [21,28]. These negative effects increase the burden of adrenal disease and should be integrated into healthcare systems, for instance, with regard to hypertension, type 2 diabetes, or osteoporosis [39,40]. However, the decision of adrenalectomy is not routinely recommended since not all complications are correctable with surgery [41]. An adequate and prompt identification in NFAs/MACS and patient stratification is based on good collaborative networking between different specialists and between primary, secondary, and tertiary healthcare divisions [42]. Hence, the importance of addressing such tumors from a multidisciplinary perspective also includes the identification of the surgery group and the integration of follow-up protocols for non-surgery candidates. As expected from many other areas of chronic conditions, predictive scores and algorithms of follow-up might help the overall management [43,44,45].

The limits of the current study include the retrospective, transversal design in a relatively small sample size, which was restricted by numerous exclusion criteria via triple stratification, which we considered essential in order to provide an adequate clinical, imaging, and endocrine analysis. Another bias might come from not performing a routine oral glucose tolerance test in the non-diabetic population. Supplementary information such as osteoporotic fracture risk assessment, particularly in menopausal women, and scales to assess the quality of life might add to the general clinical picture, help the evaluation of the disease burden, and refine long-standing management, including the cardiovascular and metabolic burden. Larger studies will contribute to the identification of “metabolically healthy MACS” [46] and those who are suitable surgery candidates, while steroids metabolomics in NFAs/MACS should be merged with radiomics for a better outcome and to avoid unnecessary investigations across patients’ life spans.

## 5. Conclusions

Adrenal incidentalomas belong to a complex scenario of detection in the modern medical era, requiring multidisciplinary networking since patients might be initially detected in different departments (as seen in the current study) and then referred to primary healthcare for further decision. Since these are consecutive patients according to the mentioned inclusion/exclusion criteria, this study confirmed general features with respect to the female-to-male ratio in NFA/MACS. A relatively higher rate of bilateral involvement was found, with a MACS prevalence of 23.33%, regardless of the uni/bilateral involvement or gender distribution. The MACS-positive profile adds to the disease burden and might require additional assessments during follow-up and a protocol of surveillance, including a tailored decision of tumor removal. Identification of an adrenal incidentaloma at CT scans and its hormonal characterization needs to be integrated into the panel of various chronic disorders of one patient, and a good collaboration between the endocrine team and primary healthcare physician might improve the overall long-term outcomes.

## Figures and Tables

**Table 1:** Demographic characteristics of the entire cohort (Abbreviations: BMI = body mass index, HBP = high blood pressure, N = number of patients, SD = standard deviation, T2D = type 2 diabetes).

Parameter	Descriptive Statistics	Value
Female	N (%)	47 (78.33)
HBP	N (%)	40 (66.67)
T2D	N (%)	17 (28.37)
Age (years)	Mean ± SD	60.72 ± 10.62
	Median (Q1, Q3)	63.00 (54.00, 67.00)
	Minimum, maximum	40.00, 82.00
BMI (kg/sqm)	Mean ± SD	30.37 ± 5.03
	Median (Q1, Q3)	29.85 (26.94, 32.91)
	Minimum, maximum	20.00, 43.63

**Table 2:** Characteristics of the adrenal incidentalomas on the entire group (Abbreviations: ACTH = adrenocorticotropic hormone, C-B = morning plasma (baseline) cortisol, C-6 pm = plasma cortisol at 6 p.m., C-1 mg-DST = second-day plasma cortisol after 1 mg dexamethasone test, UFC = 24-h urinary free cortisol, MACS = mild autonomous cortisol secretion, M = median, N = number of patients, Q = quartile, SD = standard deviation).

Parameter	Descriptive Statistics	Value
MACS-positive	N (%)	14 (23.33)
Unilateral tumor	N (%)	44 (73.33)
Right tumor	N (%)	18 (30.00)
Left tumor	N (%)	26 (43.33)
Bilateral tumors	N (%)	16 (26.67)
Largest tumor diameter (mm)	Mean ± SD	26.08 ± 8.78
	Median (Q1, Q3)	25.50 (19.00, 32.00)
	Minimum, maximum	10.00, 51.00
ACTH (pg/mL) (7.20–63.30)	Mean ± SD	19.19 ± 12.44
	Median (Q1, Q3)	14.50 (11.44, 21.00)
	Minimum, maximum	8.00, 62.44
C-B (μg/dL) (6.24–18.00)	Mean ± SD	14.55 ± 5.07
	Median (Q1, Q3)	13.90 (10.68, 18.00)
	Minimum, maximum	5.73, 34.20
C-6 pm (μg/dL) (2.69–10.40)	Mean ± SD	5.09 ± 2.34
	Median (Q1, Q3)	4.83 (3.20, 6.62)
	Minimum, maximum	1.30, 11.50
C-1 mg-DST (μg/dL) (<1.8)	Mean ± SD	1.94 ± 2.16
	Median (Q1, Q3)	1.32 (0.10, 1.79)
	Minimum, maximum	0.67, 14.50
UFC (μg/24 h) (11.50–102.00)	Mean ± SD	38.53 ± 27.51
	Median (Q1, Q3)	34.70 (21.60, 45.85)
	Minimum, maximum	9.50, 148.80

**Table 3:** Mean largest tumor diameter by age groups with 95% confidence interval (CI = confidence interval, N = number of patients, SD = standard deviation).

Age Group (Years)	N (%)	Largest Tumor Diameter (mm) Mean ± SD	95% CI	*p*-Value
40–49	10 (16.66)	28.00 ± 11.72	19.62–36.38	0.535
50–59	15 (25.00)	25.09 ± 6.05	21.75–28.44	
60–69	24 (40.00)	26.43 ± 8.54	22.83–30.04	
70–79	8 (13.33)	27.38 ± 11.19	18.02–36.73	
80–89	3 (5.00)	18.33 ± 0.58	16.90–19.77	

**Table 4:** Adrenal incidentalomas location by age groups (N = number of patients).

Age Group (Years)	Bilateral Tumors N (%)	Right Adrenal Tumor N (%)	Left Adrenal Tumor N (%)	*p*-Value
40–49	2 (20.00)	4 (40.00)	4 (40.00)	0.735
50–59	7 (46.67)	3 (20.00)	5 (33.33)	
60–69	5 (20.83)	8 (33.33)	11 (45.83)	
70–79	1 (12.50)	2 (25.00)	5 (62.50)	
80–89	1 (33.33)	1 (33.33)	1 (33.33)	

**Table 5:** Prevalence of MACS and non-MACS profile by age groups (MACS = mild autonomous cortisol secretion, N = number of patients).

Age Group (Years)	N (%)	MACS N (%)	Non-MACS N (%)	*p*-Value
40–49	10 (16.66)	2 (20.00)	8 (80.00)	0.330
50–59	15 (25.00)	4 (26.67)	11 (73.33)	
60–69	24 (40.00)	4 (16.67)	20 (83.33)	
70–79	8 (13.33)	4 (50.00)	4 (50.00)	
80–89	3 (5.00)	0 (0.00)	3 (100.00)	

**Table 6:** Correlation between adrenal incidentalomas characteristics (ACTH = adrenocorticotropic hormone, BMI = body mass index, C-B = morning plasma (baseline) cortisol, C-6 pm = plasma cortisol at 6 p.m., C-1 mg-DST = second-day plasma cortisol after 1 mg dexamethasone test, UFC = 24-h urinary free cortisol).

	Largest Tumor Diameter (mm)	ACTH (pg/mL)	C-B (μg/dL)	C-6 pm (μg/dL)	C-1 mg-DST (μg/dL)	UFC (μg/24 h)
Age (years)	*p* = 0.677 r = −0.038	*p* = 0.365 r = 0.019	*p* = 0.107 r = −0.145	*p* = 0.275 r = −0.098	*p* = 0.759 r = 0.028	*p* = 0.341 r = 0.086
BMI (kg/sqm)	*p* = 0.583 r = 0.049	*p* = 0.481 r = 0.147	***p* = 0.030** **r = −0.193**	*p* = 0.515 r = −0.058	*p* = 0.198 r = −0.114	***p* = 0.038** **r = −0.185**
Largest tumor diameter (mm)		*p* = 0.415 r = 0.183	*p* = 0.627 r = 0.044	*p* = 0.325 r = 0.088	***p* = 0.007** **r = 0.241**	*p* = 0.999 r = 0.000
ACTH (pg/mL)	*p* = 0.415 r = 0.183		*p* = 0.255 r = 0.069	*p* = 0.194 r = −0.043	*p* = 0.135 r = −0.030	*p* = 0.520 r = −0.171
C-B (μg/dL)	*p* = 0.627 r = 0.044	*p* = 0.255 r = 0.069		***p* < 0.001** **r = 0.320**	*p* = 0.623 r = −0.043	***p* = 0.011** **r = 0.226**
C-6 pm (μg/dL)	*p* = 0.325 r = 0.088	*p* = 0.194 r = −0.043	***p* < 0.001** **r = 0.320**		***p* = 0.010** **r = 0.229**	*p* = 0.108 r = 0.143
C-1 mg-DST (μg/dL)	***p* = 0.007** **r = 0.241**	*p* = 0.135 r = −0.030	*p* = 0.632 r = −0.043	***p* = 0.010** **r = 0.229**		*p* = 0.326 r = 0.087
UFC (μg/24 h)	*p* = 0.999 r = 0.000	*p* = 0.520 r = −0.171	***p* = 0.011** **r = 0.226**	*p* = 0.108 r = 0.143	*p* = 0.326 r = 0.087	

Bolded values are statistically significant.

**Table 7:** Demographic parameters of the patients with adrenal incidentalomas according to female vs. male population (BMI = body mass index, HBP = high blood pressure, N = number of patients, SD = standard deviation, T2D = type 2 diabetes).

Parameter	Descriptive Statistics	Female (N = 47, 78.33%)	Male (N = 13, 21.67%)	*p*-Value
HBP	N (%)	30 (63.82)	10 (76.92)	0.902
T2D	N (%)	13 (27.66)	4 (30.77)	0.826
Age (years)	Mean ± SD	62.40 ± 10.47	54.62 ± 9.11	**0.018**
	Median (Q1, Q3)	65.00 (55.00, 69.00)	58.00(50.00, 61.00)	
	Minimum, maximum	44.00, 82.00	40.00, 66.00	
BMI (kg/sqm)	Mean ± SD	30.77 ± 5.29	28.94 ± 3.76	0.249
	Median (Q1, Q3)	30.00 (27.80, 34.36)	28.08 (25.60, 32.50)	
	Minimum, maximum	20.00, 43.63	22.85, 34.60	

Bolded value is statistically significant.

**Table 8:** Characteristics of adrenal incidentalomas according to the patient gender (ACTH = adrenocorticotropic hormone, C-B = morning plasma (baseline) cortisol, C-6 pm = plasma cortisol at 6 p.m., C-1 mg-DST = second-day plasma cortisol after 1 mg dexamethasone test, UFC = 24-h urinary free cortisol, MACS = mild autonomous cortisol secretion, M = median, N = number of patients, Q = quartile, SD = standard deviation).

Parameter	Descriptive Statistics	Female (N = 47, 78.33%)	Male (N = 13, 21.67%)	*p*-Value
MACS-positive	N (%)	11 (23.40)	3 (23.08)	0.980
Unilateral tumor	N (%)	34 (72.34)	10 (76.92)	0.741
Right tumor	N (%)	13 (27.66)	5 (38.46)	
Left tumor	N (%)	21 (44.68)	5 (38.46)	
Bilateral tumors	N (%)	13 (27.66)	3 (23.08)	
Largest tumor diameter (mm)	Mean ± SD	26.63 ± 9.16	24.08 ± 7.23	0.357
	Median (Q1, Q3)	26.00 (19.00, 32.50)	25.00 (20.00, 28.00)	
	Minimum, maximum	13.00, 51.00	10.00, 35.00	
ACTH (pg/mL) (7.20–63.30)	Mean ± SD	20.47 ± 10.53	18.92 ± 11.28	0.444
	Median (Q1, Q3)	21.20 (13.50, 29.17)	25.06 (15.10, 32.00)	
	Minimum, maximum	11.36, 53.80	8.00, 50.37	
C-B (μg/dL) (6.24–18.00)	Mean ± SD	14.44 ± 5.41	14.95 ± 3.74	0.755
	Median (Q1, Q3)	13.60 (10.30, 18.00)	14.60 (13.00, 17.30)	
	Minimum, maximum	5.73, 34.20	8.90, 20.50	
C-6 pm (μg/dL) (2.69–10.40)	Mean ± SD	5.19 ± 2.54	4.71 ± 1.42	0.377
	Median (Q1, Q3)	4.87 (3.17, 6.86)	4.63 (4.20, 5.31)	
	Minimum, maximum	1.30, 11.50	2.21, 7.58	
C-1 mg-DST (μg/dL) (<1.8)	Mean ± SD	1.99 ± 2.41	1.76 ± 0.84	0.093
	Median (Q1, Q3)	1.20 (0.90, 1.79)	1.56 (1.34, 1.70)	
	Minimum, maximum	0.67, 14.50	1.04, 4.33	
UFC (μg/24 h) (11.50–102.00)	Mean ± SD	40.40 ± 30.04	31.75 ± 14.04	0.590
	Median (Q1, Q3)	34.70 (21.60, 46.55)	34.70 (22.10, 39.30)	
	Minimum, maximum	10.60, 148.80	9.50, 57.30	

**Table 9:** Demographic parameters of subjects with adrenal incidentalomas by unilateral/bilateral tumor location (BMI = body mass index, HBP = high blood pressure, N = number of patients, SD = standard deviation, T2D = type 2 diabetes).

Parameter	Descriptive Statistics	Unilateral (N = 44, 73.33%)	Bilateral (N = 16, 26.67%)	*p*-Value
Female	N (%)	34 (77.27)	13 (81.25)	0.741
HBP	N (%)	28 (63.64)	12 (75.00)	0.409
T2D	N (%)	13 (29.55)	4 (25.00)	0.730
Age (years)	Mean ± SD	61.41 ± 10.57	58.81 ± 10.86	0.407
	Median (Q1, Q3)	63.00 (53.00, 67.50)	57.00 (54.50, 65.00)	
	Minimum, maximum	44.00, 82.00	40.00, 80.00	
BMI (kg/sqm)	Mean ± SD	30.64 ± 4.71	29.63 ± 5.92	0.494
	Median (Q1, Q3)	30.05 (27.80, 33.30)	28.92 (24.90, 32.91)	
	Minimum, maximum	22.60, 42.50	20.00, 43.63	

**Table 10:** Adrenal incidentalomas characteristics by unilateral/bilateral location (ACTH = adrenocorticotropic hormone, C-B = morning plasma (baseline) cortisol, C-6 pm = plasma cortisol at 6 p.m., C-1 mg-DST = second-day plasma cortisol after 1 mg dexamethasone test, UFC = 24-h urinary free cortisol, MACS = mild autonomous cortisol secretion, M = median, N = number of patients, Q = quartile, SD = standard deviation).

Parameter	Descriptive Statistics	Unilateral (N = 44, 73.33%)	Bilateral (N = 16, 26.67%)	***p*-Value**
MACS-positive	N (%)	9 (20.46)	5 (31.25)	0.382
Largest tumor diameter (mm)	Mean ± SD	25.51 ± 9.25	27.65 ± 7.38	0.408
	Median (Q1, Q3)	25.00 (18.00, 30.50)	29.00 (23.00, 33.20)	
	Minimum, maximum	13.00, 51.00	10.00, 36.00	
ACTH (pg/mL) (7.20–63.30)	Mean ± SD	19.55 ± 10.96	22.67 ± 13.03	0.517
	Median (Q1, Q3)	20.59 (12.06, 28.54)	25.00 (11.32, 36.03)	
	Minimum, maximum	13.85, 62.44	12.60, 50.53	
C-B (μg/dL) (6.24–18.00)	Mean ± SD	14.20 ± 4.49	15.53 ± 6.46	0.373
	Median (Q1, Q3)	13.85 (11.20, 17.60)	14.60 (10.22, 18.20)	
	Minimum, maximum	5.73, 24.60	8.29, 34.20	
C-6 pm (μg/dL) (2.69–10.40)	Mean ± SD	4.69 ± 1.98	6.18 ± 2.94	0.075
	Median (Q1, Q3)	4.65 (3.10, 5.90)	6.00 (3.50, 8.05)	
	Minimum, maximum	1.30, 9.86	2.21, 11.50	
C-1 mg-DST (μg/dL) (<1.8)	Mean ± SD	1.65 ± 1.10	2.73 ± 3.74	0.867
	Median (Q1, Q3)	1.33 (1.00, 1.75)	1.21 (0.92, 2.49)	
	Minimum, maximum	0.67, 5.80	0.71, 14.50	
UFC (μg/24 h) (11.50–102.00)	Mean ± SD	37.79 ± 26.53	40.56 ± 30.86	0.953
	Median (Q1, Q3)	34.80 (21.60, 44.40)	32.45 (20.30, 46.10)	
	Minimum, maximum	9.50, 148.80	11.40, 134.40	

**Table 11:** Demographic parameters of subjects with adrenal incidentalomas with MACS and without MACS (BMI = body mass index, HBP = high blood pressure, MACS = mild autonomous cortisol secretion, N = number of patients, SD = standard deviation, T2D = type 2 diabetes).

Parameter	Descriptive Statistics	MACS (N = 14, 23.33%)	Non-MACS (N = 46, 76.67%)	*p*-Value
Female	N (%)	11 (78.57)	36 (78.36)	0.980
HBP	N (%)	9 (64.29)	31 (67.4)	0.625
T2D	N (%)	1 (7.14)	16 (34.78)	**0.044**
Age (years)	Mean ± SD	60.21 ± 9.76	60.87 ± 10.97	0.842
	Median (Q1, Q3)	62.00 (51.00, 70.00)	63.00 (54.00, 67.00)	
	Minimum, maximum	44.00, 74.00	40.00, 82.00	
BMI (kg/sqm)	Mean ± SD	28.07 ± 4.83	31.07 ± 4.92	**0.049**
	Median (Q1, Q3)	27.64 (25.09, 30.21)	30.05 (27.90, 33.80)	
	Minimum, maximum	20.00, 36.41	22.85, 43.63	

Bolded values are statistically significant.

**Table 12:** Characteristics of adrenal incidentalomas with MACS-positive and MACS-negative features (ACTH = adrenocorticotropic hormone, C-B = morning plasma (baseline) cortisol, C-6 pm = plasma cortisol at 6 p.m., C-1 mg-DST = second-day plasma cortisol after 1 mg dexamethasone test, UFC = 24-h urinary free cortisol, MACS = mild autonomous cortisol secretion, M = median, N = number of patients, Q = quartile, SD = standard deviation).

Parameter	Descriptive Statistics	MACS (N = 14, 23.33%)	Non-MACS (N = 46, 76.67%)	*p*-Value
Unilateral tumor	N (%)	9 (64.29)	35 (76.09)	0.382
Right tumor	N (%)	5 (35.71)	13 (28.26)	
Left tumor	N (%)	4 (28.57)	22 (47.83)	
Bilateral tumors	N (%)	5 (35.71)	11 (23.91)	
Largest tumor diameter (mm)	Mean ± SD	30.14 ± 6.81	24.84 ± 9.00	**0.047**
	Median (Q1, Q3)	32.00 (24.75, 36.00)	25.00 (18.00, 30.00)	
	Minimum, maximum	18.00, 40.00	10.00, 51.00	
ACTH (pg/mL) (7.20–63.30)	Mean ± SD	19.98 ± 9.12	21.30 ± 13.02	0.371
	Median (Q1, Q3)	22.63 (17.03, 31.19)	25.06 (17.90, 34.62)	
	Minimum, maximum	13.59, 49.99	12.68, 50.94	
C-B (μg/dL) (6.24–18.00)	Mean ± SD	15.69 ± 6.81	14.21 ± 4.44	0.342
	Median (Q1, Q3)	13.95 (10.90, 18.10)	13.90 (9.83, 17.90)	
	Minimum, maximum	8.29, 34.20	5.73, 21.50	
C-6 pm (μg/dL) (2.69–10.40)	Mean ± SD	6.40 ± 2.40	4.69 ± 2.20	**0.015**
	Median (Q1, Q3)	5.81 (4.98, 7.56)	4.26 (2.98, 5.98)	
	Minimum, maximum	2.55, 11.50	1.30, 9.86	
C-1 mg-DST (μg/dL) (<1.8)	Mean ± SD	4.38 ± 3.53	1.19 ± 0.33	**<0.001**
	Median (Q1, Q3)	2.90 (2.23, 5.45)	1.19 (0.89, 1.45)	
	Minimum, maximum	1.88, 14.50	0.67, 1.79	
UFC (μg/24 h) (11.50–102.00)	Mean ± SD	43.41 ± 31.20	37.04 ± 26.48	0.479
	Median (Q1, Q3)	35.20 (26.30, 52.60)	33.25 (21.60, 45.00)	
	Minimum, maximum	10.60, 128.40	9.50, 148.80	

Bolded values are statistically significant.

## Data Availability

All the available data are already within the paper.

## Abbreviations

The following abbreviations are used in this manuscript:

ACTH	adrenocorticotropic hormone
BMI	body mass index
CT	computed tomography
CI	confidence interval
C-B	baseline morning plasma cortisol
C-6 pm	plasma cortisol at 6 p.m.
C-1 mg-DST	second-day plasm cortisol after 1 mg dexamethasone test
HBP	high blood pressure
M	median
N	number of patients
NFAs	nonfunctioning adrenal adenomas
Q	quartile
SD	standard deviation
T2DM	type 2 diabetes mellitus
vs.	versus
UFC	24-h urinary free cortisol
